# Broadly applicable TCR-based therapy for multiple myeloma targeting the immunoglobulin J chain

**DOI:** 10.1186/s13045-023-01408-6

**Published:** 2023-02-27

**Authors:** Miranda H. Meeuwsen, Anne K. Wouters, Tassilo L. A. Wachsmann, Renate S. Hagedoorn, Michel G. D. Kester, Dennis F. G. Remst, Dirk M. van der Steen, Arnoud H. de Ru, Els P. van Hees, Martijn Kremer, Marieke Griffioen, Peter A. van Veelen, J. H. Frederik Falkenburg, Mirjam H. M. Heemskerk

**Affiliations:** 1 Department of Hematology, Leiden University Medical Center, Albinusdreef 2, 2333 ZA Leiden, The Netherlands; 2 Center for Proteomics and Metabolomics, Leiden University Medical Center, Albinusdreef 2, 2333 ZA Leiden, The Netherlands

## Abstract

**Background:**

The immunoglobulin J chain (Jchain) is highly expressed in the majority of multiple myeloma (MM), and Jchain-derived peptides presented in HLA molecules may be suitable antigens for T-cell therapy of MM.

**Methods:**

Using immunopeptidomics, we identified Jchain-derived epitopes presented by MM cells, and pHLA tetramer technology was used to isolate Jchain-specific T-cell clones.

**Results:**

We identified T cells specific for Jchain peptides presented in HLA-A1, -A24, -A3, and -A11 that recognized and lysed *JCHAIN*-positive MM cells. TCRs of the most promising T-cell clones were sequenced, cloned into retroviral vectors, and transferred to CD8 T cells. Jchain TCR T cells recognized target cells when *JCHAIN* and the appropriate HLA restriction alleles were expressed, while *JCHAIN* or HLA-negative cells, including healthy subsets, were not recognized. Patient-derived *JCHAIN*-positive MM samples were also lysed by Jchain TCR T cells. In a preclinical in vivo model for established MM, Jchain-A1, -A24, -A3, and -A11 TCR T cells strongly eradicated MM cells, which resulted in 100-fold lower tumor burden in Jchain TCR versus control-treated mice.

**Conclusions:**

We identified TCRs targeting Jchain-derived peptides presented in four common HLA alleles. All four TCRs demonstrated potent preclinical anti-myeloma activity, encouraging further preclinical testing and ultimately clinical development.

**Supplementary Information:**

The online version contains supplementary material available at 10.1186/s13045-023-01408-6.

## Background

Multiple myeloma (MM) is a malignancy of the bone marrow (BM) characterized by uncontrolled expansion of plasma cells. Advances in treatment options have extended survival of MM patients, but curative therapies with an acceptable safety profile are lacking [1]. To date, allogeneic stem cell transplantation (allo-SCT) has been the only curative therapy for MM, but allo-SCT is associated with high toxicity and treatment-related mortality. Recently, B-cell maturation antigen (BCMA) targeting chimeric antigen receptor (CAR) T cells have extended treatment options for MM patients. BCMA is expressed in a subset of memory B cells and in plasma cells as well as in MM. BCMA CAR T cells were effective and safe, but long-term complete responses were rare [2–4]. Relapses often resulted from heterogenous BCMA expression within tumors, which led to outgrowth of antigen-low or negative MM cells [5, 6], suggesting that targeting of a single antigen may be insufficient to induce durable complete responses in a majority of patients. A curative approach for treating multiple myeloma will likely require multi-antigen targeting. Alternative CAR targets like SLAMF7 and GPRC5D are currently being explored, but safety and efficacy of targeting these antigens remain to be proven [7, 8]. The requirement for CAR targets to be present on the cell surface largely restricts discovery of new CAR target antigens. Therefore, T-cell receptor (TCR)-engineered T cells could be of additional value since TCRs can recognize peptides presented in human leukocyte antigen (HLA) that can be derived from any protein. This includes antigens derived from intracellular proteins that would be inaccessible for conventional CAR T cells, thereby accessing an additional pool of potential MM antigens.

Recently, we identified the immunoglobulin J chain (Jchain) as a new target antigen for MM [9]. *JCHAIN* is highly expressed in the majority of patient MM samples whereas expression in healthy tissues of non-B-cell origin is absent [9]. Physiologically, the Jchain protein links monomers in multimeric IgA and IgM when secreted by plasma cells. Furthermore, Jchain facilitates transport of dimeric IgA and pentameric IgM across mucosal barriers, where poly-Ig receptors bind to Jchain on the basolateral side of the membrane after which the complexes are secreted on the luminal side [10]. Despite its functional role in multimerization of IgA and IgM, *JCHAIN* expression appears to be independent of the isotype as Jchain is also expressed in plasma cells secreting other isotypes [11, 12]. *JCHAIN* expression was previously described in MM, and, importantly, *JCHAIN* expression was maintained with disease progression even when immunoglobulin production was lost [13]. Similar to plasma cells, *JCHAIN* expression in MM was independent of the immunoglobulin isotype produced [14]. These observations suggest Jchain as a promising antigen for T-cell-based targeting of MM. The intracellular expression of Jchain precludes CAR-mediated recognition and would require TCR-mediated targeting of Jchain-derived epitopes presented in the context of HLA on MM cells. Since Jchain-derived peptides are non-mutated peptides, high-affinity T cells recognizing Jchain epitopes presented in self-HLA molecules are deleted during thymic development [15]. To circumvent this immunogenic tolerance, the immunogenicity of self-peptides in foreign HLA molecules can be exploited using HLA mismatched donors [9, 16–21].

In this study, we identified Jchain-derived epitopes presented by MM cells in HLA-A*01:01 (HLA-A1), HLA-A*02:01 (HLA-A2), HLA-A*03:01 (HLA-A3), HLA-A*11:01 (HLA-A11) and HLA-A*24:02 (HLA-A24). Respective peptide-HLA (pHLA) tetramers were generated and used to isolate Jchain-specific T cells from peripheral blood mononuclear cells (PBMCs) of HLA mismatched healthy donors [9, 22]. T-cell clones recognizing peptides presented in HLA-A1, HLA-A3, HLA-A11, and HLA-A24 were identified. Upon sequencing and transfer of TCRs from selected T-cell clones, Jchain-specific TCR-transduced CD8 T cells demonstrated potent killing of MM in vitro as well as in vivo*,* while no off-target effects were observed. Therefore, the TCRs identified in this study hold great promise for therapy of MM.

## Methods

### JCHAIN microarray data

Log2 transformed expression values of *JCHAIN* (probe: 212592_at) and *SDC1* (probe: 201286_at) were retrieved from publicly available microarray dataset (GSE: 13,591; https://www.ncbi.nlm.nih.gov/geo/geo2r/?acc=GSE13591) [23]. The dataset consists of expression profiles of 133 multiple myeloma patient samples and 5 healthy donor plasma cell samples (sorted on CD138 +). Expression values were back-transformed using the function: *y* = 2^*x*. The cutoff for expression was defined using a selection of genes not expressed in MM (*GFAP*_203540_at, *INS*_ 206598_at, *MUC16*_ 220196_at, *MYH11*_ 201496_x_at, *TRAC*_ 209670_at) calculated as mean values of the selected negative genes + (3×STDEV).

### JCHAIN expression

*JCHAIN* expression was measured by quantitative real-time polymerase chain reaction (qPCR) as previously described [9]. *JCHAIN* forward primer: 5′ GTACCATTTGTCTGACCTCTGT 3′ and reverse primer: 5′ AGCAGGTCTCTGTAGCACTG 3′. *GUSB*, *VPS29*, and *PSMB4* were included as housekeeping genes (HKGs). *JCHAIN* expression was calculated relative to HKGs.

### Epitope discovery

MM cell lines U266 (HLA-A*02:01, -A*03:01, -B*07:02, -B*40:01, -C*03:04, and -C*07:02) and UM9 (HLA-A*01:01, -A*11:01, -B*07:02, -B*55:01, -C*03:04, and -C7*07:02) were used for Jchain epitope discovery. U266 cells were retrovirally transduced with HLA-A24 and UM9 cells were transduced with HLA-A2. Retroviral transduction was performed as previously described [24]. pHLA-class I complexes were isolated from MM cell lysates from 30 × 10^9 cells using anti-HLA class I (Clone W6/32) or an anti-HLA-A1/A24 (Clone GV5D1, provided by Dr. Arend Mulder, Leiden University Medical Center, the Netherlands) antibodies after which peptide elution, mass spectrometry analysis and epitope selection were performed as previously described [9]. Peptide elution data were investigated for peptides originating from the Jchain protein according to the UniProt *Homo sapiens* database. Peptides were assigned to HLA class I (HLA-I) alleles expressed by cells of origin for which the peptides contain anchor residues for binding the respective HLA allele according to netMHC4.0 [25]. Mass spectra of identified peptides were compared to mass spectra of synthetically generated peptides to confirm correct identification. Peptide-HLA monomers were refolded, and successful refolding was used as criterium for stable binding of identified peptides to designated HLA-I molecules.

### Cell culture and target cell generation

T cells were cultured in TCM consisting of IMDM (Lonza) supplemented with 5% fetal bovine serum (FBS; Gibco, Life Technologies), 5% human serum 1.5% glutamine (Lonza) and 1% penicillin/streptomycin (Lonza) and 100 IU/ml IL2 (Proleukin; Novartis Pharma). Expanded T-cell clones were cryopreserved or restimulated every 10–15 days with a feeder mix containing 1 × 10^6 PBMC’s, 0.1 × 10^6 Epstein–Barr virus-transformed lymphoblastoid cell line (EBV-LCL) JY cells and 0.8 mg/ml phytohemagglutinin (PHA; Oxoid Microbiology Products, Thermo Fisher Scientific). Cell lines were cultured in IMDM with 10% FBS, 1.5% glutamine and 1% penicillin/streptomycin. Cell lines negative for target HLA alleles were retrovirally transduced to express HLA-A1, -A24, -A3 or -A11. Viral vectors encoded murine CD19 or tNGFR as transduction markers. K562 cells were additionally transduced to express *JCHAIN*. Transduced cells were purified for transgene expression by MACS enrichment of transduction marker positive cells. PBMCs from healthy donors expressing one or two target HLA alleles were used to derive hematopoietic subsets as previously described [22]. Fibroblasts and keratinocytes were pre-treated for 48 h with 100 IU/ml IFN-*γ* (Immukine) prior to experiments. MM patient-derived BM samples were thawed and rested overnight in medium containing 10% human serum before use in cytotoxicity experiments.

### T-cell assays

pHLA tetramer-sorted T cells were initially screened for peptide reactivity in a high-throughput manner as previously described [9]. Peptide reactive T-cell clones were selected and screened for recognition of endogenously processed and presented peptide using *JCHAIN* transduced K562 cells. T-cell clones producing > 2 ng/ml IFN-y were selected for further investigation. For cytokine production experiments, 5,000 T cells were co-cultured with 30,000 target cells. IFN-y production after overnight co-culture was measured by ELISA (R&D systems). In peptide titration experiments, target cells were loaded with decreasing peptide concentrations starting at 1uM. Supernatants were tested in 5 × and 125 × dilution. For tetramer binding experiments, T cells were stained with 2 ug/ml PE-labeled Jchain pHLA tetramers and analyzed by fluorescence-activated cell sorting (FACS).

### Cytotoxicity experiments

Killing of cell lines was tested in standard ^51^chromium release assays as described previously [9]. Killing of primary material was analyzed in FACS-based cytotoxicity assays. 50,000 target cells were co-cultured with T cells in effector/target (E/T) ratio 3:1 overnight. After overnight culture cells were stained with specific antibody panels and SYTOX Blue Dead Cell Stain (Invitrogen by Thermo Fisher Scientific) was added prior to acquisition to identify living cells. Samples were acquired by FACS using fixed flow rates and stable acquisition was validated using Flow-Count Fluorospheres (Beckman Coulter). Co-cultures with activated B cells were stained using anti-CD3 (Alexa Fluor 700, BD Pharmingen), anti-CD19 (PE-Cy7, BD Pharmingen), anti-IgA (PE, Miltenyi), anti-IgG (FITC, DAKO) and anti-IgM (APC, BD). For primary MM, 50,000 patient-derived BM mononuclear cells were used as target cells. Co-cultures were stained with anti-CD3, anti-CD19 (APC, BD Pharmingen), anti-CD45 (FITC, BD), anti-CD38 (PE-Texas Red, Invitrogen), and anti-CD56 (PE-Cy7, BD Pharmingen). MM cells were defined as CD3^neg^, CD19^neg^, CD45^neg−int^, CD38^pos^, CD56^pos^.

### TCR sequencing and transfer

TCR*α* and TCR*β* sequences of selected T-cell clones were identified as previously described [9]. TCR*α* and *β* variable chains were codon-optimized and introduced into MP71-TCR-flex retroviral vectors encoding cysteine-modified murine TCR*αβ* constant domains. CD4 and CD8 T cells were separately isolated, activated, and transduced with TCR as previously described [9]. On day 7 post-activation, TCR-transduced T cells were enriched for expression of introduced TCRs using indirect MACS and APC labeled anti-mouse TCR-C*β* (mTCR; BD Pharmingen). Purified T cells were used in experiments between day 10 and 14 after activation.

### pHLA tetramer-based T-cell isolations

PE-labeled pHLA tetramers were produced as previously described with minor modifications [9, 26]. PBMCs isolated from complete buffy coats (Sanquin) obtained from healthy donors negative for HLA-I alleles of interest were used to isolate Jchain-specific T-cell clones as previously described [9]. In short, PBMCs were incubated with pooled pHLA tetramers and pHLA tetramer bound cells were enriched by MACS using anti-PE MicroBeads (Miltenyi Biotec). Enriched fractions were single-cell sorted for pHLA tetramer^pos^ CD8^pos^ T cells in 96-well round-bottom plates containing feeder mix consisting of 5 × 10^5 irradiated PBMCs, 5 × 10^4 irradiated EBV-LCL JY cells and 0.8 mg/ml PHA in T-cell medium (TCM).

### In vivo* MM model*

U266 cells were transduced with and enriched for Luciferase-tdTomato Red and target HLA-A alleles when indicated. NOD-scid-IL2Rgamma^null^ (NSG) mice (The Jackson Laboratory) were intravenously (i.v.) injected with 2 × 10^6 U266 cells. After 21 days, mice were treated i.v. with 3–6 × 10^6 purified Jchain TCR-transduced CD8 T cells. CMV TCR T cells were used as negative control. Tumor outgrowth was measured at regular intervals after subcutaneous (s.c.) injection of 150 µL 7.5 mM D-luciferine (Cayman Chemical) using a CCD camera (IVIS Spectrum, PerkinElmer). All mice were killed when control mice reached an average luminescence of 1 × 10^7 p/s/cm^2^/sr. This study was approved by the National Ethical Committee for Animal Research (AVD116002017891) and performed in accordance with Dutch laws for animal experiments. Statistical analysis was performed using GraphPad Prism version 9.3.1. In vivo experiments were analyzed using 2-way ANOVA comparing groups per timepoint with Sidak’s multiple comparisons post hoc test.

## Results

**Fig. 1:**
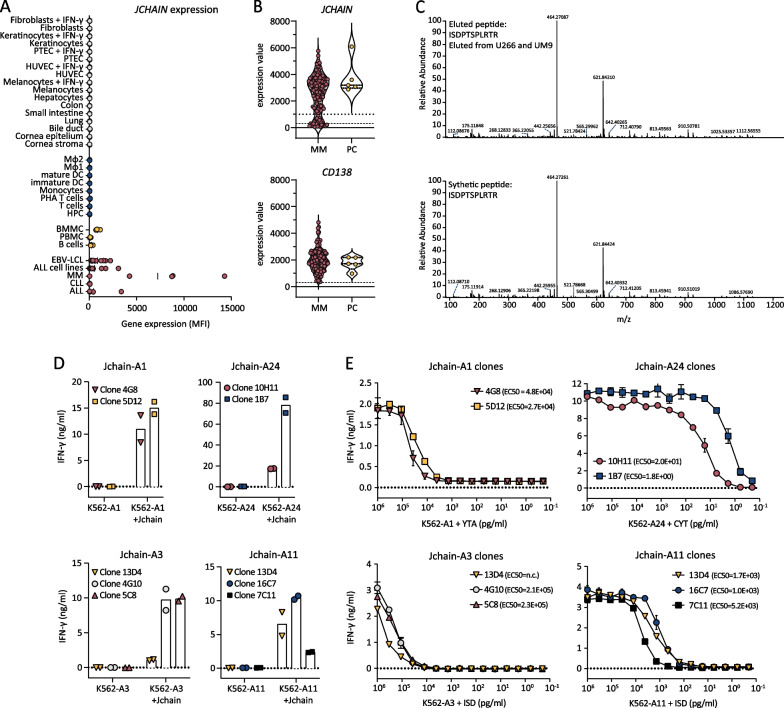
Jchain target identification and subsequent selection of promising T-cell clones recognizing Jchain peptides in HLA-A1, A24, A3 and A11. **A** *JCHAIN* microarray data (probe ILMN_2105441) from an Illumina HT12.0 microarray dataset (GSE76340) displaying gene expression levels in mean fluorescence intensities (MFI) in healthy tissue of non-hematopoietic origin (in gray), healthy tissue of hematopoietic origin (in blue), healthy subsets containing B cells (yellow) and B- and plasma cell malignancies (in red). Per healthy tissue gene expression was measured in 2–7 samples (mean; 3.4). Abbreviations: ALL, acute lymphoblastic leukemia; CLL, chronic lymphocytic leukemia; MM, multiple myeloma; EBV-LCL, Epstein–Barr virus-transformed lymphoblastoid cell lines; PBMC, peripheral blood mononuclear cells; BMMC, bone marrow mononuclear cells; HPC, hematopoietic precursor cells; DC, dendritic cells; MΦ1, type 1 macrophages; MΦ2, type 2 macrophages; IFN-*γ*, interferon-*γ*; HUVEC, human umbilical vein endothelial cells; PTEC, proximal tubular epithelial cells. **B** Violin plot of *JCHAIN* expression (top graph) in 133 MM samples and 5 healthy plasma cell (PC) samples. Samples were enriched for CD138; therefore, CD138 (*SCD1*) expression is displayed as positive control (bottom graph). Data were extracted from publicly available dataset GSE13591. Dashed lines indicate background level for negative expression values. Dotted line indicates an arbitrary cutoff for samples expressing *JCHAIN* (expression value > 1000). **C** Example of verification of peptide identification by mass spectrometry. Tandem mass spectra of ISDPTSPLRTR peptide eluted from HLA-A3 positive U266 MM cells as well as HLA-A11 positive UM9 MM cells (top graph). Corresponding tandem mass spectra of synthetic ISDPTSPLRTR peptide (bottom graph). **D, E** IFN-*γ* production by Jchain-specific T cells clones after overnight co-culture measured by ELISA. Graphs are separated based on peptide-HLA specificities. Averages of technical duplicates are depicted. **D** Jchain T-cell clones co-cultured with K562 target cells transduced with target HLA alleles (-A1, -A24, -A3, or –A11) without or with additional transduction of the *JCHAIN* gene (+ Jchain). **E** T-cell clones overnight stimulated with antigen negative K562 cells transduced with target HLA molecules HLA-A1, -A24, -A3 or -A11 loaded with decreasing concentrations of Jchain peptides
- *Identification of Jchain-specific T-cell clones from HLA mismatched healthy donors*

- *JCHAIN as target gene for MM and epitope discovery by mass spectrometry*
  Previously, we identified target genes for cellular therapy of B-cell malignancies using Illumina microarray data [9, 27]. *JCHAIN* was identified as promising target since it was highly expressed in 4 out of 5 MM patient samples, and expression in healthy tissues of non-B-cell origin was absent (Fig. 1A). To gain insight in the fraction of MM patients that could benefit from Jchain targeting therapy, gene expression data of 133 MM patient samples were extracted from a publicly available microarray dataset (GSE13591) [23]. 81% of samples were clearly positive for *JCHAIN* expression (expression value > 1000) (Fig. 1B). To discover epitopes for T cell targeting of the Jchain, pHLA-I complexes were isolated from *JCHAIN* positive MM cell lines U266 and UM9 cells (Additional file 1: Fig. S1A) and peptides eluted from HLA-I were analyzed by mass spectrometry. Mass spectrometry data were analyzed for peptides originating from the Jchain protein according to the UniProt *Homo sapiens* database. To determine HLA-I origin of identified Jchain peptides, peptides were assessed for the presence of anchor residues for binding to HLA-I alleles expressed by U266 and UM9 using netMHC4.0 [25]. Peptides with anchor residues for commonly expressed HLA-A alleles: HLA-A1, -A2, -A3, -A11, or -A24 were selected. This resulted in identification of 8 candidate peptides (Table 1). Peptide, ISDPTSPLRTR, was presented in HLA-A3 as well as HLA-A11. For the candidate peptides, correct sequence identification was confirmed by comparing mass spectra of eluted peptides to mass spectra of synthetic peptides (Fig. 1C). For all 9 assigned peptide-HLA combinations pHLA monomers could be refolded, indicating that the peptides bind their respective HLA-I alleles despite variable predicted binding affinities (Table 1).

**Table 1:** Jchain-derived peptides identified by peptide elution and mass spectrometry from MM cell lines UM9 or U266 presented in HLA-A1, -A2, -A3, -A11 or -A24

	Sequence	Jchain aa	Eluted from	HLA-I^c^	%Rank_EL^d^	WB/SB^e^	HLA binding
		Position^a^	MM cell line^b^				Confirmed^f^
YTA-A1	YTAVVPLVY	132–140	UM9	A1	0.02	SB	Yes
TAV-A1	TAVVPLVY	133–140	UM9	A1	1.30	WB	Yes
VLA-A2^1^	VLAVFIKAVHV	10–20	U266	A2	3.22	< WB	Yes
VLA-A2^2^	VLAVFIKAV	10–18	UM9 and U266	A2	0.23	SB	Yes
YTA-A2	YTAVVPLV	132–139	UM9	A2	4.17	< WB	Yes
ISD-A3	ISDPTSPLRTR	72–82	U266	A3	3.16	< WB	Yes
RII-A11	RIIVPLNNR	61–69	UM9	A11	0.24	SB	Yes
ISD-A11	ISDPTSPLRTR	72–82	UM9	A11	1.90	WB	Yes
CYT-A24	CYTAVVPLV	131–139	U266^g^	A24	0.96	WB	Yes

^a^Amino acid (aa) position of identified peptides within the Jchain protein according to UniProt

^b^UM9 cells (HLA-A1, -A11, -B7, -B35, -C3 and -C7 positive) were HLA-A2-transduced and U266 cells (HLA-A2, -A3, -B7, -B40, -C3, and -C7 positive) were HLA-A24-transduced

^c^Most likely HLA-I origin of peptides based on HLA typing of MM cells from which peptides were eluted and peptide-binding motifs of these HLA alleles according to NetMHC4.0

^d^Rank of the predicted binding score for the eluted peptide to the respective HLA molecule compared to a set of random natural peptides

^e^Peptides were annotated as weak or strong binders using the netMHC4.1 default setting of 0.5% rank for strong binders (SB) and 2% rank for weak binders (WB). Peptides with %Rank > 2.0 were annotated as < WB

^f^Peptide binding to the respective HLA allele was investigated by peptide-HLA monomer refolding. Yes: peptide-HLA monomers were successfully refolded and remained stable. No: peptide-HLA monomers could not stably be refolded

^g^Epitope identified in HLA-peptide elution experiment using anti-HLA-A1/A24 antibody but not in elution using pan HLA-I antibody W6-32
  To identify Jchain-specific T-cell clones of high avidity, we exploited T-cell immunogenicity in an HLA-mismatched setting [9]. PBMCs from donors that are negative for the HLA restriction alleles of the target peptides were incubated with PE-labeled pHLA tetramers (Table 1) followed by single-cell sorting and clonal expansion of pHLA tetramer^pos^ CD8^pos^ T cells. In total, 26 buffy coats were used from which 17,000 T-cell clones could be expanded. Peptide-specific T-cell clones were identified by high-throughput screenings as previously described using the HLA-negative myeloid leukemia cell line K562 transduced with target HLA alleles loaded with Jchain peptides [9]. In additional screenings, T-cell clones were tested for recognition of *JCHAIN* and HLA-transduced K562 cells to identify clones that are able to recognize endogenously processed and presented peptide. Based on recognition of endogenous antigen the 9 most potent T-cell clones were selected. T-cell clones 4G8 and 5D12 recognized Jchain peptide YTAVVPLVY in HLA-A1 (YTA-A1), T-cell clones 10H11 and 1B7 recognized CYTAVVPLV in HLA-A24 (CYT-A24) and T-cell clones 13D4, 4G10, 5C8 16C7 and 7C11 recognized ISDPTSPLRTR in HLA-A3 (ISD-A3) or in HLA-A11 (ISD-A11) (Fig. 1D). Of note, T-cell clone 13D4 recognized ISD-A3 as well ISD-A11. For the other epitopes no peptide-specific T-cell clones were identified, or recognition of *JCHAIN* transduced cells was low or absent. The avidities of the identified T-cell clones for target peptides YTA-A1, CYT-A24, ISD-A3 and ISD-A11 were tested in peptide titration experiments. T-cell clones that efficiently recognized *JCHAIN* transduced K562 cells exhibited different avidities between peptide specificities with average EC50 values ranging from 2.2 × 10^5 pg/ml for ISD-A3 to 1.1 × 10^1 pg/ml for YTA-A24 (Fig. 1E). Per specificity, 2–3 promising T-cell clones were identified, from which the two most potent T-cell clones were selected for follow-up screenings of safety and potency. Given the weak recognition of ISD-A3 but strong recognition of ISD-A11, clone 13D4 was further analyzed for recognition of HLA-A11 positive targets.
- *Safety screenings revealed stringent on-target recognition of T-cell clones*
  As TCRs identified from the HLA mismatched repertoire are often promiscuous, specificity of selected T-cell clones needs to be assessed to identify potentially harmful cross-reactivities. First, recognition of look-alike peptides presented in the target HLA alleles was investigated using a panel of *JCHAIN* negative (Additional file 1: Fig. S1B), target HLA allele positive cell lines from multiple tissue origins (Fig. 2A). Allo-HLA T-cell clones, recognizing peptides derived from housekeeping proteins presented in respective HLA molecules, were used to verify that targets were generally susceptible to T-cell recognition (Additional file 1: Fig. S2). None of the Jchain-specific T-cell clones revealed aberrant reactivity to *JCHAIN*-negative cells expressing target HLA restriction alleles. As Jchain T-cell clones showed no off-target recognition when the target HLA is present, we continued to explore whether T-cell clones would cross-react with other HLA alleles. T-cell clones were stimulated with a panel of EBV-LCLs designed to express as many common HLA-I alleles as possible without expressing the target restriction allele (Fig. 2B, Additional file 1: Table S1). For Jchain-A1, both T-cell clones showed no recognition of any of the EBV-LCLs. For both Jchain-A24 and Jchain-A3, one clone per specificity showed recognition of multiple EBV-LCLs, leading to the exclusion of these T-cell clones. For Jchain-A11, both T-cell clones weakly recognized EBV-LCL VJY. While this could indicate cross-reactivity to an HLA allele expressed by EBV-LCL VJY, recognition could alternatively be mediated by presentation of the Jchain-derived ISD peptide presented in a look-alike HLA allele. Indeed, EBV-LCL VJY expresses HLA-A*36:01 for which Jchain ISD is predicted to be a weak binder [25]. Based on the recognition profiles, we continued with T-cell clones 5D12 (Jchain HLA-A1), 10H11 (Jchain HLA-A24), 5C8 (Jchain HLA-A3), and 16C7 (Jchain HLA-A11) as these clones demonstrated acceptable safety profiles and highest potencies.

**Fig. 2:**
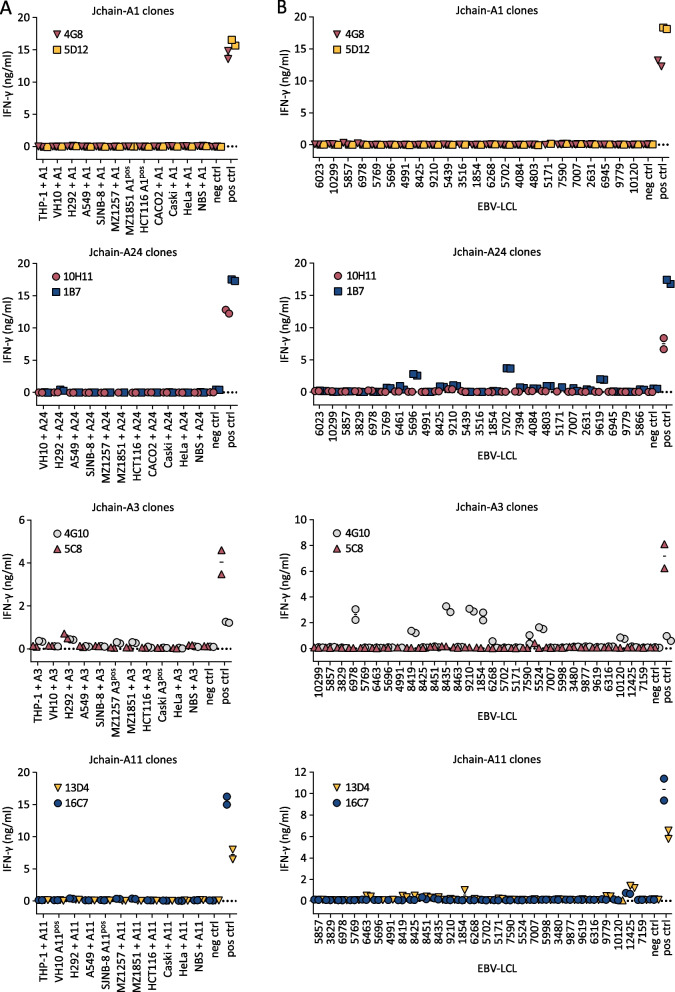
Investigation of cross-reactivity by Jchain-specific T-cell clones. IFN-*γ* production by T-cell clones measured by ELISA after overnight co-culture, technical duplicates are depicted. Graphs are separated based on peptide-HLA specificities. **A** T-cell clones were stimulated with a panel of cell lines of non-B cell origins transduced with target HLA (+ A1, A24, A3 or A11) or naturally expressing target HLA molecules (A1^pos^, A24^pos^, A3^pos^ or A11^pos^). HLA-transduced K562 cells were included as negative control (neg ctrl), target gene and HLA-transduced K562 cells were included as positive control for T-cell function (pos ctrl). THP-1 A24 was not recognized by allo-HLA-A24 T-cell clone; therefore, this cell line was excluded from data as stimulatory capacity is lacking. **B** T-cell clones were stimulated with an EBV-LCL panel containing EBV-LCLs expressing HLA-I alleles with an allele frequency over 1% that do not express target HLA alleles. Controls as in **A**
- *Jchain-specific T-cell clones potently recognize and lyse MM cell lines*
  To determine the ability of the selected Jchain T-cell clones to target MM cells, recognition and killing of MM cell line U266 was assessed. HLA-A1 and HLA-A24 Jchain-specific T-cell clones 5D12 and 10H11 specifically lysed HLA-A1 or HLA-A24 transduced U266 cells, respectively (Fig. 3A). Jchain HLA-A3 T-cell clone 5C8 lysed WT HLA-A3^pos^ U266 cells, as well as U266 cells additionally transduced to express HLA-A11, while Jchain HLA-A11 T-cell clone 16C7 only recognized U266 transduced with HLA-A11 (Fig. 3A). Target cell lysis by Jchain-specific clones reached similar levels as allo-HLA T-cell clones that were used as positive controls. Additionally, target cell lysis by Jchain-specific T-cell clones concurred with IFN-*γ* production upon co-culture (Fig. 3B). Combined, these data demonstrate that Jchain-specific T-cell clones recognized and lysed *JCHAIN* expressing MM cells in an HLA-dependent manner.
- *TCR gene transfer installs Jchain-specific effector functions onto CD8 T cells*
  To investigate the potential for TCR gene transfer, TCRs of Jchain-specific T-cell clones were sequenced, cloned into retroviral expression vectors, and introduced into CD4 and CD8 T cells. For all four TCRs, introduction into healthy donor CD8 T cells followed by TCR enrichment resulted in functional TCR expression as demonstrated by pHLA tetramer binding (Fig. 4A). Staining intensity was lower in CD8 T cells compared to parental T-cell clones. TCR 16C7 (ISD-A11) transduced CD4 T cells also bound pHLA tetramer, but at a lower level than CD8 T cells demonstrating dependence on the CD8 co-receptor for optimal pHLA tetramer binding. To investigate the functionality of Jchain TCR-transduced T cells, Jchain TCR T cells were co-cultured with *JCHAIN* transduced K562 cells. Jchain TCR CD8 T cells produced cytokine when co-cultured with K562 cells transduced to express *JCHAIN* and the respective target HLA, but not when cultured with *JCHAIN* negative K562 cells (Fig. 4B). Functionality of Jchain TCR-transduced CD4 T cells was absent or limited compared to CD8 T cells, and Jchain TCR-transduced CD4 T cells were therefore not investigated further. Lytic potential of Jchain TCR-transduced CD8 T cells was studied using *JCHAIN* expressing MM cell lines U266 and UM9, and *JCHAIN*-negative K562 cells as negative control. Jchain HLA-A1, -A24, -A3, and -A11 TCR-transduced CD8 T cells induced potent lysis of *JCHAIN* ^pos^ U266 as well as UM9 cells, while antigen negative K562 cells were not lysed (Fig. 4C). Lysis of target cells was accompanied by antigen-specific cytokine production of TCR-transduced CD8 T cells (Additional file 1: Fig. S3). In conclusion, Jchain TCR-transduced CD8 T cells demonstrated Jchain-specific effector functions.

**Fig. 3:**
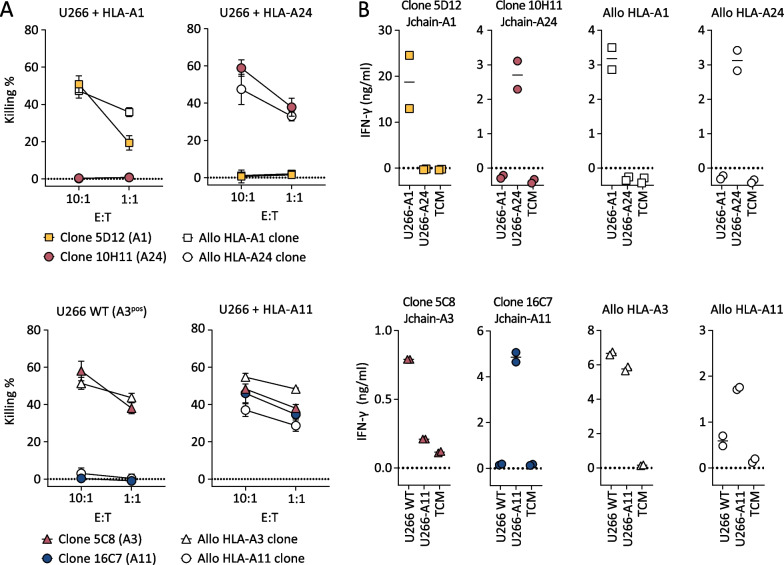
Killing of MM cell line U266 by Jchain-specific T-cell clones. **A** Killing of *JCHAIN* expressing U266 cells transduced with HLA-A1 or A24 by Jchain A1 and A24 specific T-cell clones in a 6-h 51Cr release assay (top graphs). Killing of WT (HLA-A3 positive) or HLA-A11-transduced U266 cells by Jchain HLA-A3 or A11-specific T-cell clones (bottom graphs). Killing assays were performed using E/T ratios 10:1 and 1:1. Allo-HLA T-cell clones, recognizing peptides derived from housekeeping proteins in specific HLA alleles, were included as positive controls. Average values and standard deviations of technical triplicates are shown. **B** IFN-*γ* production measured by ELISA after overnight co-culture using the same target cells as in **A** in an E/T of 1:6. Values and means of technical duplicates are shown
- *Jchain TCR T cells target healthy B cells but not JCHAIN negative healthy tissues*
  To assess the safety profile of Jchain TCR T cells, Jchain TCR-transduced T cells were co-cultured with healthy subsets of hematopoietic and non-hematopoietic origin. Hematopoietic subsets including immature dendritic cells (DCs), mature DCs, PHA-activated T cells, and B cells were tested. Fibroblasts and keratinocytes were used as non-hematopoietic cell types. Per healthy subset, cells from multiple donors positive or negative for HLA-A1, -A3, -A11, and -A24 were included. Stimulatory capacity of healthy subsets was confirmed upon recognition by allo-HLA T-cell clones (Additional file 1: Fig. S4) and *JCHAIN* expression was measured by qPCR (Additional file 1: Fig. S1C). Jchain TCR-transduced CD8 T cells did not recognize *JCHAIN* negative (< 0.1 relative to HKG) primary cell subsets, but *JCHAIN* expressing peripheral blood B cells were recognized when target HLA alleles were expressed (Fig. 5A). To investigate if recognition of B cells results in lysis, Jchain A1 and Jchain A24 TCR T cells were co-cultured with B cells derived from multiple donors. FACS analysis revealed specific lysis of B cells positive for target HLA alleles (Fig. 5B, C). These data demonstrate that Jchain TCR T cells maintained Jchain-specific reactivity as previously seen for parental T-cell clones. No off-target reactivity of *JCHAIN*-negative heathy cell subsets was apparent, but on-target/off-tumor reactivity of *JCHAIN* expressing B cells revealed that Jchain TCR gene therapy would likely result in depletion of the healthy B-cell compartment in vivo.

**Fig. 4:**
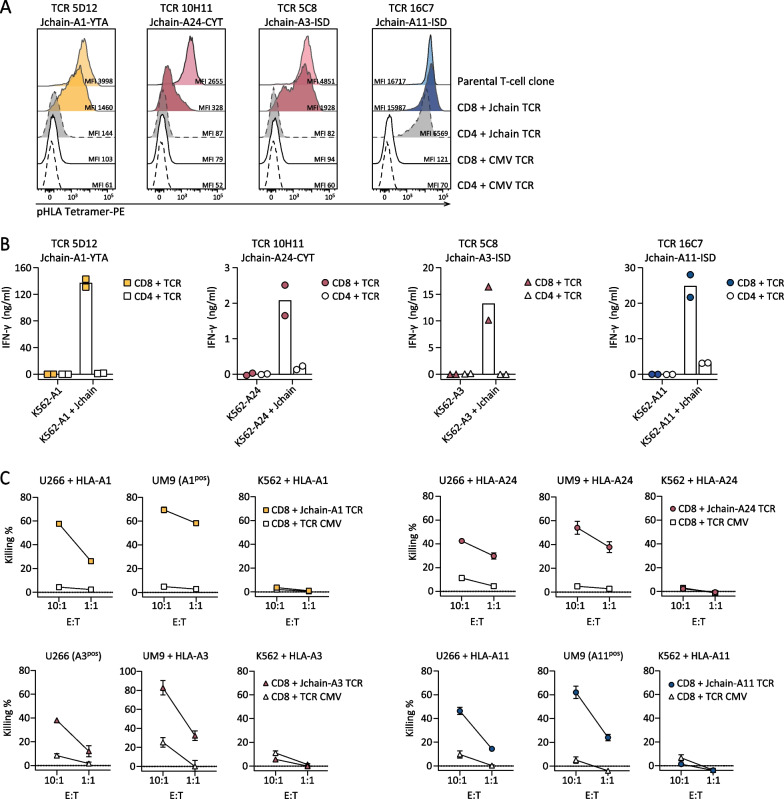
Functionality of Jchain TCRs in CD8 and CD4 T cells. **A–C** Jchain A1, A24, A3 and A11 restricted TCRs were sequenced and introduced with murine constant beta domains (mTCR) into CD4 and CD8 T cells. After mTCR enrichment functionality was assessed. **A** TCR T cells were stained with the respective Jchain pHLA tetramers and analyzed by FACS. TCR T cells were gated on mTCR +. Parental T-cell clones were included as positive controls and CMV TCR T cells were included as negative controls. **B** Endogenous recognition of *JCHAIN* and HLA (A1, A24, A3, or A11) transduced K562 cells by Jchain TCR CD4 and CD8 T cells. HLA only transduced K562 cells were included as negative control. Values and means of technical duplicates are shown. **C** TCR-transduced CD8 T cells were used for 6-h chromium release assays to study target cell lysis in E/T ratios 10:1 and 1:1. T cells were co-cultured with U266 MM cells, UM9 MM cells or antigen negative K562 cells transduced with (+ HLA) or naturally expressing (HLA^pos^) target HLA molecules. CMV TCR T cells were used as a negative control. Values and means of technical triplicates are shown
- *Jchain TCR T cells lyse patient bone marrow-derived MM cells*
  To analyze ex vivo lysis of primary MM cells, FACS-based killing assays were performed using BM samples from MM patients. MM cell frequencies in BM samples varied between 13.3% and 85.4% (Additional file 1: Fig. S5). *JCHAIN* expression in positive MM samples was ninefold to 416-fold higher than housekeeping genes (HKG), while 2 out of 8 included MM samples did not express *JCHAIN* (< 0.1 relative to HKG) (Additional file 1: Fig. S1D). MM samples were incubated overnight with Jchain HLA-A1 and -24, or Jchain HLA-A3 and -A11 TCR T cells or CMV TCR T cells as negative controls. Jchain TCR T cells efficiently lysed primary MM cells, as exemplified by co-culture of Jchain HLA-A1 and -A24 TCR T cells with BM cells from HLA-A1^pos^, A24^pos^ patient MM.J2 (Fig. 6A). All tested Jchain TCR T cells induced lysis of Jchain^pos^ target HLA allele expressing MM cells, while *JCHAIN* ^neg^ samples or samples negative for HLA restriction alleles were not lysed (Fig. 6B). However, *JCHAIN* ^pos^ MM cells from HLA-A24^pos^ patient MM.J3 and patient MM.J4 were not lysed by Jchain A24 TCR T cells (Fig. 6B), while the allo-HLA-A24 T-cell clone induced potent lysis of these samples (data not shown), demonstrating that the CYT-A24 epitope is not always presented by MM cells at levels sufficient to mediate lysis by the TCR T cells.

**Fig. 5:**
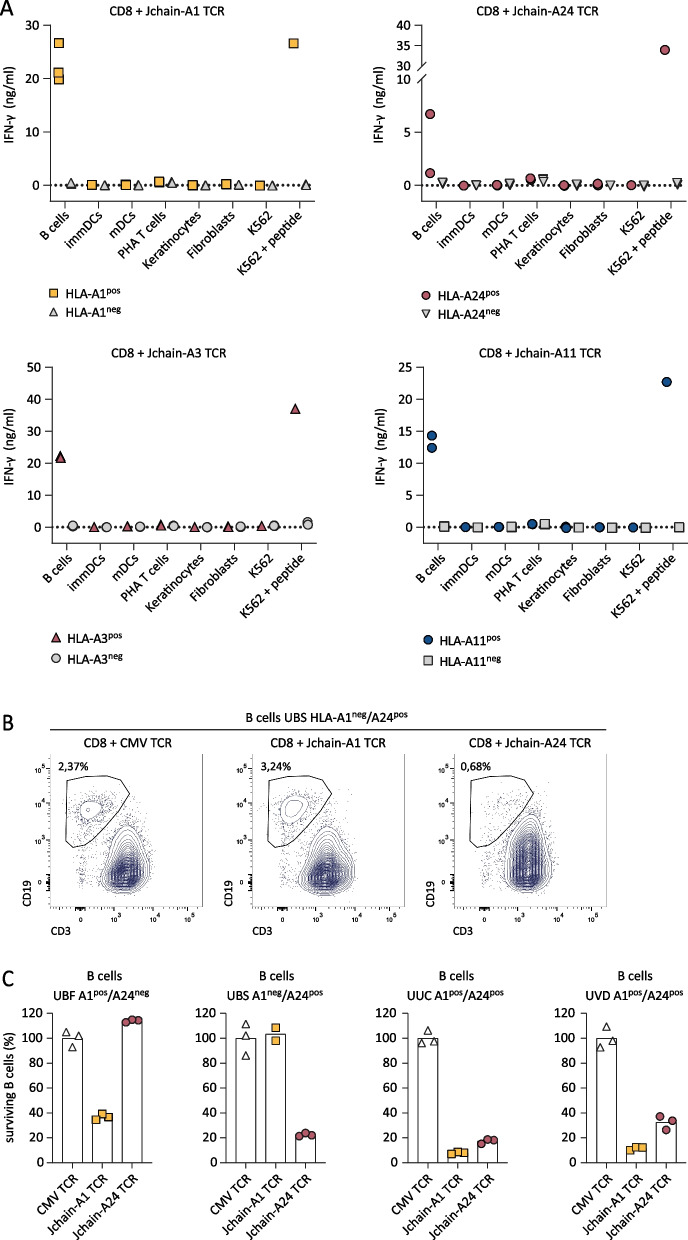
Recognition of healthy hematopoietic and non-hematopoietic subsets by Jchain TCR-transduced CD8 T cells. **A** IFN-*γ* production after overnight co-culture of Jchain TCR Td CD8 T cells with CD40L activated B cells, immature dendritic cells (immDCs), mature dendritic cells (mDCs), PHA-activated T cells (PHA T cells), and keratinocytes or fibroblasts pre-treated for 48 h with 100 IU/ml IFN-*γ*. Symbols represent the average value (from technical duplicates) of target cells isolated from different donors. Target cells not expressing the relevant HLA restriction allele are depicted in gray, cells expressing the HLA restriction alleles are depicted in color. Per panel T cells with one of the Jchain TCRs are shown as indicated in the graph titles. K562 + HLA and peptide loaded K562 + HLA are included as negative and positive controls. **B**, **C** FACS-based killing experiment of CD40L activated peripheral blood B cells from healthy donors with Jchain A1 and Jchain A24 TCR T cells in an E/T ratio of 3:1, samples were measured using fixed acquisition times and fixed flow rates. **B** Example of B cell survival and killing after overnight co-culture of HLA-A1^neg^/HLA-A24^pos^ B cells with CMV TCR CD8 T cells (left), Jchain A1 TCR T cells (middle) and Jchain A24 TCR T cells (right). Gated on SYTOX blue-, single cells, CD3-, CD19 + . **C** Quantification of percentage surviving B cells of data in **B** and additional donors, HLA-A1 and -A24 typing is indicated in graph titles. Percentage surviving cells was calculated relative to cells in the negative control CMV TCR T-cell culture. Technical triplicates are shown
- *Jchain TCR T cells potently eradicate established MM in a preclinical xenograft model*
  To test whether Jchain TCR T cells would convey in vivo activity, we used a MM xenograft model. NSG mice were inoculated with WT HLA-A3^pos^ U266 cells or HLA-A1, HLA-A24, or HLA-A11-transduced U266 cells. Tumor cells were allowed to grow for 3 weeks to model established MM. On day 21, mice were infused with Jchain HLA-A1, -A24, -A3 or -A11 TCR or irrelevant CMV TCR-transduced CD8 T cells (Fig. 7). All four Jchain TCRs substantially reduced MM tumor burden up to 6 days after infusion. Compared to control mice, tumor load in treated mice was approximately 100-fold lower, demonstrating strong in vivo anti-tumor responses (Fig. 7B*).* To conclude, our data show that identified Jchain A1, A3, A11, and A24 TCRs demonstrate potent anti-MM responses when transferred to CD8 T cells, both in vitro against patient-derived primary MM samples and in vivo against *JCHAIN* ^pos^ MM cell line U266.

**Fig. 6:**
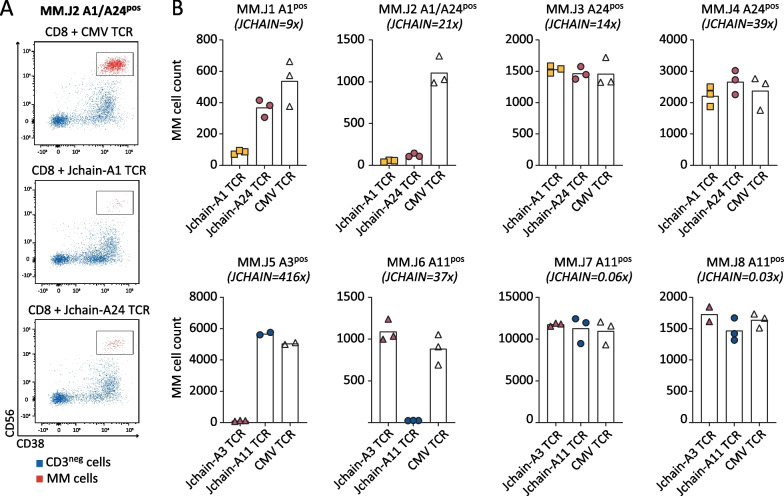
Killing of MM cells in patient-derived bone marrow samples. Killing of patient-derived bone marrow MM samples was assessed by FACS-based cytotoxicity experiments in which TCR Td T cells were co-cultured with patient BM samples in an E/T ratio 3:1. MM cell survival was analyzed after overnight co-culture. **A** Example of survival of an HLA-A1^pos^/A24^pos^ patient sample after co-culture with CD8 T cells transduced with a CMV (negative control), Jchain HLA-A1 or Jchain HLA-A24 restricted TCR. In red MM cells are displayed, highlighted by black boxes for clarity, MM cells were gated on: live cells → single cells → CD3-negative cells to exclude co-cultured T cells → CD45 negative-intermediate, CD19 negative → CD56 positive, CD38 positive. MM cells (in red) were backgated on total CD3-negative cells (in blue). **B** MM.J1-MMJ.10 codes in graph titles represent different MM patients, additionally expression of target HLA molecules and *JCHAIN* expression as a fold increase relative to housekeeping genes is displayed. *JCHAIN* expression was measured by qPCR on sorted MM cells. Numbers of surviving MM cells acquired per 2500 Flow-Count Fluorospheres are displayed. Jchain HLA-A1 and HLA-A24 TCR Td T CD8 T cells co-cultured with MM patient samples from different individuals expressing HLA-A1, A24 or both (top row). Jchain HLA-A3 and HLA-A11 TCR Td T CD8 T cells co-cultured with MM patient samples from different individuals expressing HLA-A3 or A11 (bottom row). Technical triplicates are shown. Data representative of two independent experiments

**Fig. 7:**
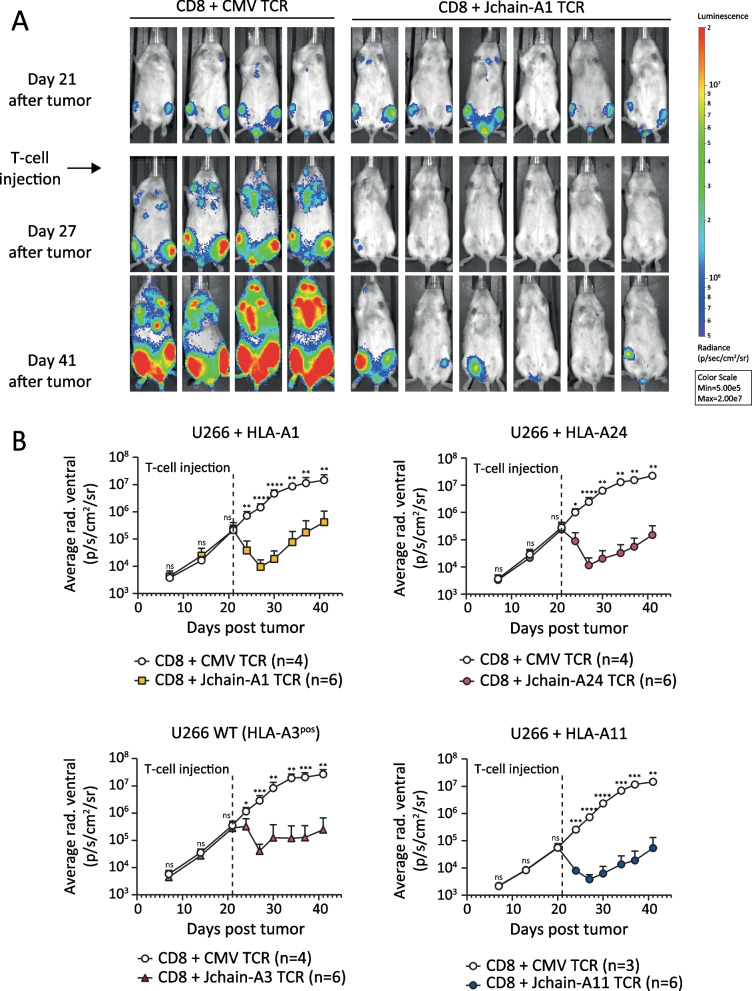
In vivo anti-tumor efficacy of Jchain HLA-A1, A24, A3 and A11 restricted TCR-transduced CD8 T cells. NSG mice engrafted with 2 × 10^6^ U266 multiple myeloma cells transduced with *Luc2* luciferase and HLA-A1, -A11, -A24 or wildtype (WT) were i.v. injected with 3–6 × 10^6^ TCR-transduced CD8 T cells after 21 days. CD8 T cells were separately transduced with Jchain HLA-A1 (5D12), -A3 (5C8), -A11 (16C7), -A24 (A24) or control CMV (pp65-NLV-HLA-A2) TCR and enriched for mTCR expression by MACS. T cells were infused 10 days after re-stimulation. Tumor outgrowth was frequently tracked by bioluminescence imaging. Data representative of two independent experiments. **A** Raw bioluminescent images of Jchain HLA-A1 TCR-treated mice and control CMV TCR-treated mice. **B** Mean and standard deviations of tumor signal (average radiance) over time on the ventral side in Jchain HLA-A1 TCR-treated (upper left), Jchain HLA-A3 TCR-treated (lower left), Jchain HLA-A24 TCR-treated (upper right) and Jchain HLA-A11 TCR-treated (lower right) mice compared to CMV TCR control mice. Statistics depict two-way ANOVA comparing groups per timepoint with Sidak’s multiple comparisons post hoc test

## Discussion

BCMA targeting CAR T-cell therapy can induce deep remissions in relapsed/refractory MM but durable responses remain rare [28]. Multi-antigen targeting of MM is a suitable approach to tackle antigen heterogeneity and immune escape in the context of cellular therapy. Alternative MM antigens that are being explored as targets for CAR therapy are CD38, SLAMF7, and CD138; however, on-target off-tumor expression of these antigens on other hematopoietic or non-hematopoietic cells compromises their safety profile. CD38 is expressed on activated lymphocytes and on a subset of CD34 + hematopoietic stem cells [29]. Similarly, SLAMF7 is expressed on a subset of T cells, and conclusively T cells engineered to target SLAMF7 undergo limited levels of fratricide [30]. CD138, a general marker for MM, is also expressed on other lymphocytes as well as some epithelial cells [31]. More recently, GPRC5D was suggested as an additional surface antigen with an expression profile more comparable to that of BCMA [8]. A first clinical trial with GPRC5D targeting CAR T cells demonstrated clinical activity and, similarly to the experience with BCMA targeting CAR T cells, relapse was associated with antigen loss [32]. Here, we present Jchain as a promising additional target with an attractive expression profile, i.e., expression is strictly limited to B- and plasma cells as well as MM. Intracellular expression of Jchain precludes CAR-mediated targeting and therefore requires TCR-mediated targeting of Jchain-derived peptides presented in HLA.

Using immunopeptidomics, pHLA tetramer technology and by exploiting the immunogenicity of non-mutated epitopes in foreign HLA alleles, we successfully identified specific T-cell clones recognizing Jchain peptides in the context of HLA-A1, -A24, -A3 and -A11. Jchain directed recognition was maintained upon TCR sequencing and transfer to CD8 T cells. Jchain TCR T cells did not recognize healthy tissue subsets other than B cells. Potent eradication of MM cells from patient-derived BM was observed for 6 out of 8 *JCHAIN* expressing MM samples. Finally, we demonstrated that Jchain TCR T cells were able to largely reduce MM tumor burden in an in vivo xenograft model. Combined, the HLA-A1, -A24, -A3 and -A11 Jchain-specific TCRs result in an HLA allele coverage of 60.7% of the average worldwide population, allowing for a broadly applicable TCR therapy for the treatment of MM.

Epitope selection in this study was performed based on HLA-peptide elution studies using MM cell lines to ensure targeting of epitopes that are naturally processed and presented on MM rather than relying solely on prediction algorithms. Despite this, a discrepancy between *JCHAIN* expression and killing of HLA-A24 positive MM cells by Jchain TCR T cells was observed, indicating that the CYT-A24 epitope is not sufficiently presented on all primary materials for TCR T-cell recognition. Similar mechanisms might play a role in presentation of the Jchain YTA-A1, ISD-A3, and ISD-A11 epitopes, but could have remained undetected since limited primary materials were tested in this study. Investigation of Jchain epitope presentation in a larger MM cohort will be needed to explore mechanisms of aberrant Jchain CYT-A24 epitope presentation and to clarify the proportion of patients that would benefit from clinical development of Jchain TCRs. For clinical application, it will be essential to include diagnostics tools to ensure Jchain epitope presentation in addition to *JCHAIN* and respective HLA expression prior to therapy of MM patients.

Safety aspects of transgenic T-cell therapy remain a major concern since both off-target and on-target/off-tumor reactivities can result in lethal toxicity [33, 34]. While off-target reactivity screenings were performed in this study, cross-reactivities might have remained undetected, and a more detailed mapping of potential cross-reactivities using peptide library scanning should be performed in additional preclinical studies. Besides off-target toxicity, on-target/off-tumor toxicity is a crucial safety aspect of engineered T-cell therapy. *JCHAIN* expression profile strongly overlaps with the expression profile of *BCMA* (proteinatlas.org version 21.0) [35, 36]. While Jchain has not been explored as a therapeutic target for MM therapy, BCMA CAR T-cell therapy has demonstrated safe application. BCMA CAR T-cell therapy was well tolerated, but *BCMA* expression in healthy plasma cells resulted in plasma cell aplasia, thereby compromising humoral immunity [37]. While off-target toxicity can be subjective to affinities of the individual CAR or TCR used [38], side effects due to on-target/off-tumor toxicity observed after BCMA CAR T might be of predictive value on the potential off-tumor effects exerted by Jchain TCR T cells.

To conclude, we identified promising Jchain TCRs targeting epitopes presented in HLA-A1, -A24, -A3, and -A11 resulting in a total HLA-allele coverage of more than 60%. TCR-transduced CD8 T cells demonstrated stringent specificity for *JCHAIN* expressing target cells with the relevant HLA restriction alleles. Potent eradication of MM cells in vitro as well as in vivo demonstrates high promise of Jchain TCR T cells for clinical development as a novel therapy of MM.

## Supplementary Information

**Additional file 1.** Supplementary data.

## Data Availability

The datasets generated and/or analyzed during the current study are available from the corresponding author upon reasonable request.

## Abbreviations

Jchain: Immunoglobulin J chain
MM: Multiple myeloma
BM: Bone marrow
CAR: Chimeric antigen receptor
allo-SCT: Allogeneic stem cell transplantation
BCMA: B-cell maturation antigen
TCR: T-cell receptor
HLA: Human leukocyte antigen
HLA-I: HLA class I
pHLA: Peptide-HLA
pHLA-I: Peptide-HLA class I
HLA-A1: HLA-A*01:01
HLA-A2: HLA-A*02:01
HLA-A3: HLA-A*03:01
HLA-A11: HLA-A*11:01
HLA-A24: HLA-A*24:02
PBMCs: Peripheral blood mononuclear cells
qPCR: Quantitative real-time polymerase chain reaction
HKGs: Housekeeping genes
FBS: Fetal bovine serum
MACS: Magnetic-activated cell sorting
FACS: Fluorescence-activated cell sorting
E/T: Effector/target
EBV-LCL: Epstein–Barr virus-transformed lymphoblastoid cell line
PHA: Phytohemagglutinin
NSG: NOD-scid-IL2Rgamma^null^
i.v.: Intravenously
s.c.: Subcutaneous
YTA-A1: Jchain peptide YTAVVPLVY presented in HLA-A1
CYT-A24: Jchain peptide CYTAVVPLV presented in HLA-A24
ISD-A3: Jchain peptide ISDPTSPLRTR presented in HLA-A3
ISD-A11: Jchain peptide ISDPTSPLRTR presented in HLA-A11
DCs: Dendritic cells
