# Supplementary material for: Broadly applicable TCR-based therapy for multiple myeloma targeting the immunoglobulin J chain
Source: J Hematol Oncol. 2023 Feb 27;16:16. doi: 10.1186/s13045-023-01408-6 (PMC9969645; doi:10.1186/s13045-023-01408-6)
Supplement: Supplementary file 1 — Additional file 1. Supplementary data. [file 13045_2023_1408_MOESM1_ESM.pdf]

Supplemental figures

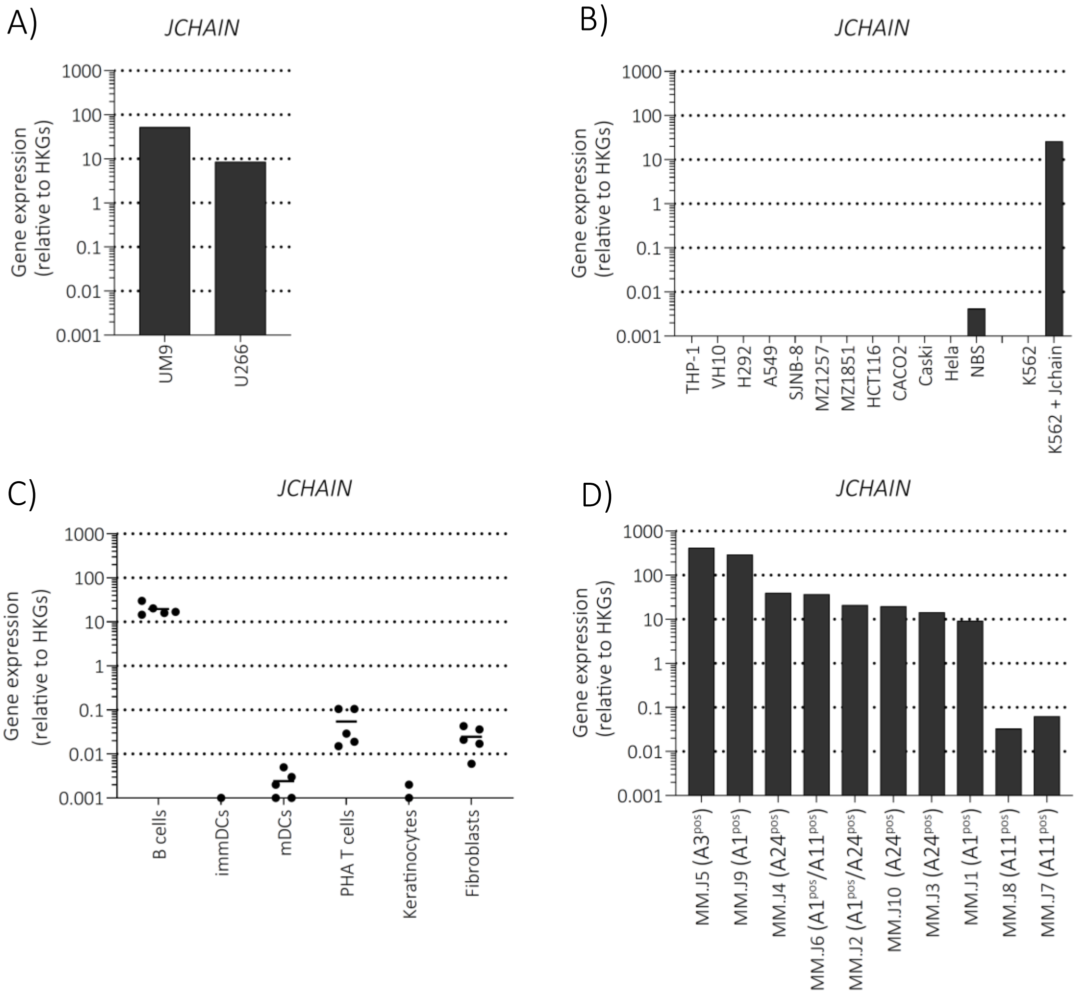

Figure s1: qRT-PCR of *JCHAIN* expression relative to house keeping genes (HKGs) in cells used in this study. **A)** MM cell lines U266 and UM9. **B)** Cell lines of non-B cell origin including K562 cells and *JCHAIN* transduced K562 cells. **C)** Healthy tissues of various origins used in figure 5 each dots represents cells from another donor. **D)** Expression in MM cells sorted from various MM patient bone marrow samples. A selection of these materials was used in figure 6.

Table S1: HLA typing of EBV-LCLs used in EBV-LCL panels

	Included in Jchain EBV-LCL panel				HLA typing		
EBV-LCL	HLA-A1	HLA-A24	HLA-A3	HLA-A11	HLA-A	HLA-B	HLA-C
6023	x	x			A*03:01/03:03/03:04 - A*11:01/11:02/11:03	B*40:02/40:35/40:37 - B*56:01	C*01:02/01:06/01:07 - C*02:02/02:04/02:08
10299	x	x	x		A*02:01 - A*11:01	B*44:05 - B*51:01	C*02:02 - C*14:02
5857	x	x	x	x	A*30:04 - A*68:02	B*38:01 - B*55:01	C*03:03 - C*12:03
3829		x	x	x	A*01:01 - A*68:01	B*44:02 - B*44:02	C*05:01 - C*07:04
6978	x	x	x	x	A*02:01 - A*02:05	B*15:01 - B*45:01	C*01:02 - C*06:02
5769	x	x	x	x	A*02:01 - A*68:01	B*35:03 - B*37:01	C*04:01 - C*06:02
6461		x			A*02:01 - A*02:01	B*40:02 - B*40:02	C*02:02 - C*02:02
6463			x	x	A*02:01 - A*02:01	B*57:01 - B*57:01	C*06:02 - C*06:02
5696	x	x	x	x	A*02:05 - A*02:05	B*58:01 - B*58:01	undetermined
4991	x	x	x	x	A*26:01/26:08/26:02 - A*31:01/31:02/31:06	B*14:01 - B*49:01	C*07:01/07:05/07:06 - C*08:02/08:07
8419			x	x	A*02:01 - A*01:01	B*50:01 - B*07:02	C*07:02 - C*06:02
8425	x	x	x	x	A*23:01 - A*02:01	B*41:01 - B*40:01	C*17:01 - C*03:04
8451			x	x	A*02:01 - A*01:01	B*35:01 - B*08:01	C*07:01 - C*04:01
8435			x	x	A*25:01 - A*02:01	B*44:02 - B*07:02	C*07:02 - C*05:01
8463			x		A*11:01 - A*01:01	B*51:01 - B*50:01	C*15:02 - C*06:02
9210	x	x	x	x	A*02:01 - A*02:01	B*15:01 - B*51:01	C*03:03 - C*15:02
5439	x	x			A*03:01 - A*25:01	B*15:17 - B*18:01/18:03/18:05	C*07:01/07:05/07:06 - C*12:03/12:06
3516	x	x			A*03:01 - A*26:01	B*07:02 - B*14:01	C*07:02 - C*08:02
1854	x	x	x	x	A*02:01 - A*30:02	B*15:01 - B*39:01	C*03:03 - C*12:03
6268	x		x	x	A*02:01 - A*24:02	B*35:02 - B*44:02	C*04:01 - C*05:01
5702	x	x	x	x	A*32:01 - A*68:01	B*35:03 - B*52:01	C*12:02 - C*12:03
7394		x			A*01:01 - A*32:01	B*35:08 - B*35:08	C*04:01 - C*04:01
4084	x	x			A*03:01/03:03/03:04 - A*30:01	B*07:02 - B*38:01	C*07:02/07:03/07:05 - C*12:03/12:06
4803	x	x			A*03:01 - A*33:01	B*07:02 - B*14:02	C*07:02 - C*08:02
5171	x	x	x	x	A*02:01 - A*66:01/66:04	B*40:01/40:11/40:14 - B*41:02	C*03:04/03:08/03:09 - C*17
7590	x		x	x	A*24:02 - A*31:01	B*07:02/07:61 - B*35:08	C*04:01 - C*07:02
5524			x	x	A*02:01/02:07/02:09 - A*31:01/31:02/31:06	B*15:01/15:33/15:34 - B*15:17	C*03:04/03:08/03:09 - C*07:01/07:05/07:06
7007	x	x	x	x	A*02:05 - A*29:02	B*27:05 - B*44:03	C*01:02 - C*16:01
2631	x	x			A*02:01 - A*03:01/03:03N/03:04	B*44:02 - B*57:01	C*06:02 - C*07:04/07:12/07:11
5998			x	x	A*24:02 - A*68:02	B*14:02 - B*38:01	C*08:02 - C*12:03
3480			x	x	A*26:01 - A*01:01	B*38:01 - B*18:01	C*12:03 - C*07:01/07:06
9877			x	x	A*01:01 - A*23:01/23:17	B*08:01 - B*41:02	C*07:01 - C*17:03
9619		x	x	x	A*01:01 - A*33:03	B*44:03 - B*51:01	C*07:06/07:18 - C*14:02
6945	x	x			A*03:01 - A*25:01	B*18:01 - B*35:01	C*04:01 - C*12:03
6316			x	x	A*29:02 - A*30:01	B*13:02 - B*44:03	C*06:02 - C*16:01
9779	x	x		x	A*02:01 - A*03:01	B*08:01 - B*50:01	C*06:02 - C*07:01
10120	x		x	x	A*02:01 - A*24:02	B*07:02 - B*40:02	C*02:02 - C*07:02
12425			x	x	A*23:01 - A*36:01	B*15:03 - B*53:01	C*02:10 - C*04:01
7159			x	x	A*02:01 - A*02:01	B*13:02 - B*44:02	C*05:01 - C*06:02
5866		x			A*02:01 - A*11:01	B*35:01 - B*51:01	C*04:01 - C*14:02

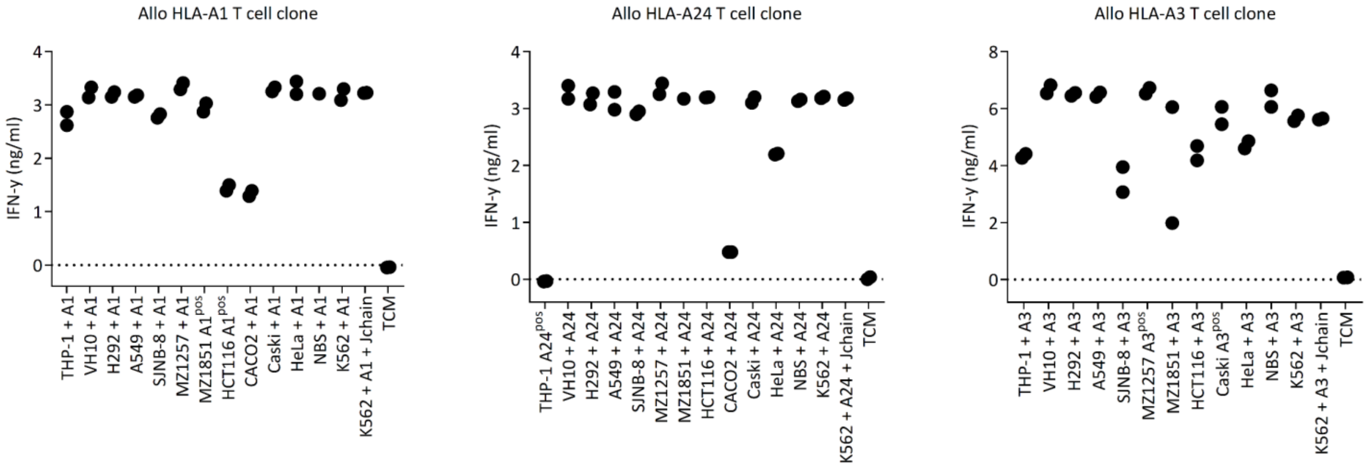

Figure s2: T cell stimulatory capacity of non-B cell lines used in safety panel. Tumor cell lines used in figure 2A co-cultured with HLA-A1 (left graph), HLA-A24 (middle graph) or HLA-A3 (right graph) allo HLA reactive T cell clones. IFN-γ concentration in supernatant was tested by ELISA using a 5-fold dilution only.

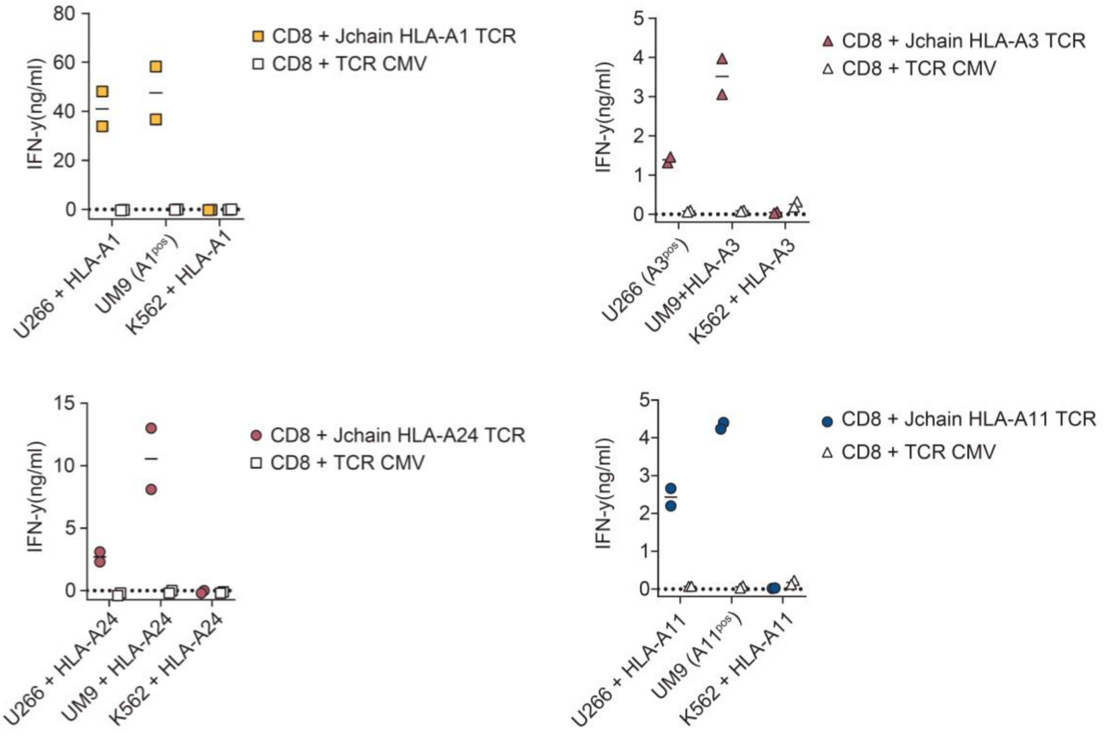

Figure S3. Antigen specific killing of MM cells by Jchain TCR CD8 T cells is associated with cytokine production. IFN-γ production measured after overnight co-culture of T cells and target cells used in figure 4C, data obtained in the same experiment. CMV TCR transduced CD8 T cells were used as a control for background cytokine production.

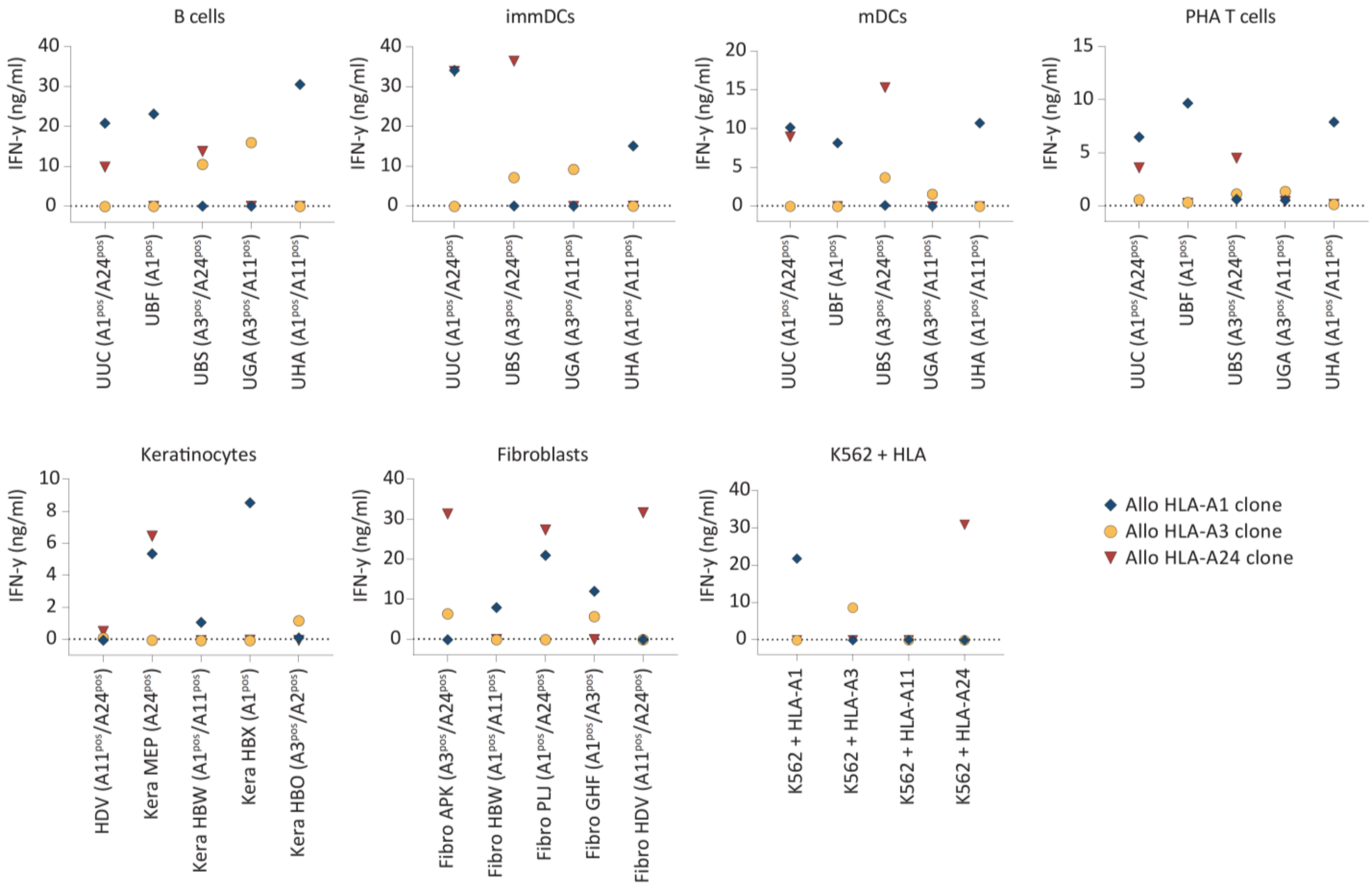

Figure s4. IFN-γ production after co-culture of Allo HLA-A1 (in blue), -A3 (in yellow) and -A24 (in red) T cell clones with all target cells used in figure 5. Three letter codes represent target cell donors and expression of HLA-A1, -A3, -A11 or -A24 is indicated. Data points represent means of technical duplicated. Graphs are separated based on cell type.

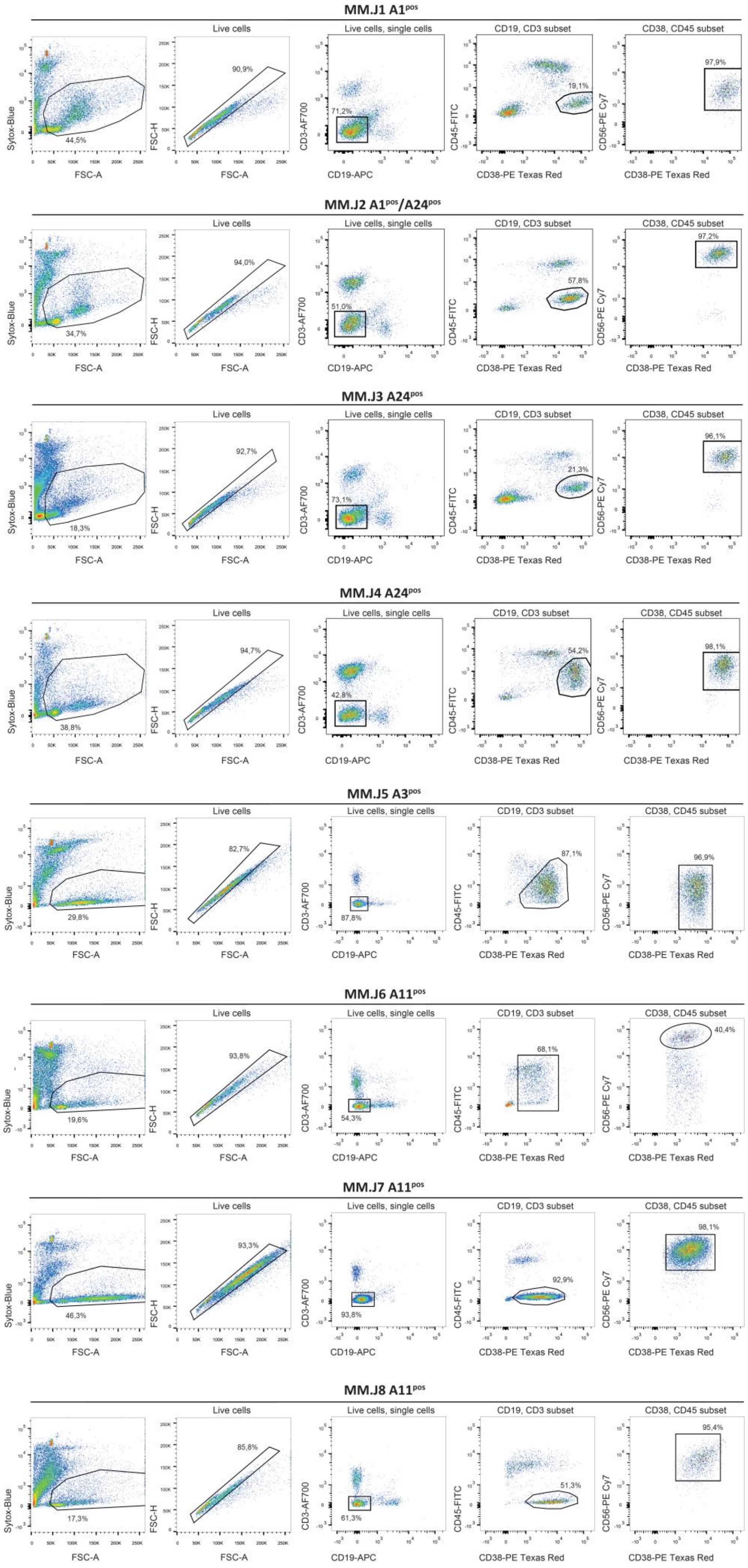

Figure S5: Phenotype of patient derived MM samples analyzed by FACS. Data obtained from the target cell only condition of the experiment shown in figure 6. Three letter codes in titles represent different patients and expression of HLA-A1, -A3, -A11 or A24 is indicated. MM patient BM samples were stained with SYTOX blue Dead Cell Stain, anti-CD3, anti-CD19, anti-CD45, anti-CD38 and anti-CD56. MM cells were gated on: SYTOX blue negative, live cells → single cells → CD3 negative, CD19 negative → CD38 positive, CD45 negative-intermediate → CD56 positive. Percentages depicted indicated the percentage of cells of the parent gate. To calculate the frequency of MM cells in BM samples, percentage in subsequent gates were multiplied.
